# Improving performance of electric vehicle drive system based a five-phase PMSM under fault using ANN and MPC

**DOI:** 10.1038/s41598-025-28210-3

**Published:** 2025-11-29

**Authors:** Ahmed M. Hassan, Mohamed Eladly Metwally

**Affiliations:** 1 Department of Electrical Power and Machines Engineering, Faculty of Engineering, Benha University, Shoubra, Cairo Egypt; 2 Department of Electrical Power and Machines Engineering, Higher Institute of Engineering (HIE), El-Shorouk Academy, El-Shorouk City, Egypt

**Keywords:** Five-Phase PMSM, Optimization techniques, Model predictive control, Artificial neural network, Electric vehicles, Online tuning, Energy science and technology, Engineering, Mathematics and computing

## Abstract

This study presents an advanced speed tracking control strategy designed to handle open-phase motor faults in EV drive systems. The proposed control strategy is evaluated using two distinct drive cycles. A five-phase interior permanent magnet synchronous motor is employed due to its notable advantages, including high efficiency, reliability, power density, and inherent fault tolerance. The control strategy leverages a multi-layer perceptron artificial neural network for online tuning of proportional-integral controller gains, enabling adaptive performance. This approach is benchmarked against a recent metaheuristic optimization algorithm known as Honey Badger. To further enhance performance, model predictive control is applied using a tailored cost function to minimize current harmonics and torque ripple. Simulation results, conducted in MATLAB Simulink, validate the effectiveness of the proposed method. Compared to conventional technique, the new approach achieves lower values for motor torque ripples and speed percentage overshoot, mean square error and integral absolute error across both test drive cycles. Additionally, the proposed method gives lower THD and attains energy saving.

## Introduction

Electric vehicles (EVs) are an important alternative to traditional fuel vehicles to reduce air pollution^1,2^. To increase the reliability of the EV, an enhanced fault-tolerant control method should be introduced in the EV drive system. The main component of the EV drive system is the electric motor. The interior permanent magnet synchronous motor (IPMSM) is commonly used in the EV drive system because of its many benefits^2,3^. These benefits include excellent reliability, lowest losses, high power density, etc^2,4,5^. Multiphase motors are becoming a promising alternative to 3-phase motors owing to reduced torque ripples and improved fault tolerance^6,7^. Model Predictive Control (MPC) is an increasingly advanced control strategy for motor drives, offering optimized and enhanced performance^8^. The analytical modeling capabilities of motor drive systems make them particularly well-suited for MPC applications. This control method effectively handles multiple operating conditions and constraints, allowing it to prioritize each one based on its significance to the overall system. The fault-tolerant control (FCT) system in an electric vehicle is crucial for maintaining stability and ensuring effective performance when a fault occurs. According to the authors’ knowledge, there are no published studies that have investigated the fault-tolerant control of an electric vehicle drive system, subjected to motor open phase fault (OPF), using a 5-phase IPMSM, model predictive control, and Artificial Neural Network online tuning, specifically designed for various drive cycles. The purpose of the presented control is to enhance the speed tracking response and to reduce energy consumption during motor open phase faults.

Literature surveys of control techniques either in healthy or faulty mode regarding the adopted drive system are explored as follows:

A sensorless control for a 5-ph PMSM was introduced in^9^. A direct torque MPC method for a 5-phase PMSM was dealt with in^10–12^. In^13^ a model-free predictive control approach was presented to leverage an ultra-local model and the outputs of motor for 5-phase PMSM drive system. Reference^14^ suggested an MPC approach for a five-phase PMPSM that employs voltage vector pre-selection.

In^15^ an FTC-based MPTC technique with harmonic tuning and pre-select voltage for a 5-ph PMSM drive system under OPF. Sensorless FTC methods for a 5-phase PMSM were presented in^16,17^, in the case of OPF. In^18^ an FTC-based MPC control method for a 5-phase PMSM was introduced. An active FTC integrated with an extended Kalman filter observer and backstepping controller for EV speed tracking driven by 5-phase PMSM in case of sensor failure was presented in^19^. Reference^20^ presented the FTC method based on decoupling control and current compensation for a 5-phase PMSM with independent neutrals. A deadbeat predictive current FTC approach for 6-phase PMSM, in the case of OPF, was presented in^21^. Reference^22^ introduced an FTC-based MPC control method for a 5-phase PMSM in the case of OPF and multiple OPFs. FTC considered reluctance torque was presented in^23^ for a 5-phase PMSM to overcome the torque ripples resulting from the reluctance torque in case of fault. An FTC based on MPC was presented in^24^ for a 5-phase PMS hub motor under OPF and two OPFs. An FTC-based FOC for a 5-phase PMSM was presented in^25^ in the case of OPF. An indirect correction approach of imaginary vectors for a 5-phase PMSM FTC under OPF was presented in^26^.

Several optimization methods were studied in^27^ to determine the best values of the gains for the PI controller in a 3-phase PMSM drive. Different control methods were presented in^28–31^ for a 3-phase PMSM using MPC-based ANN. Reference^32^ presented a control approach using a BP ANN for a 3-phase PMSM. Sensorless control of a PMSM using an adaptive speed observer was presented in^33^. Reference^34^ introduced an MPC approach for a 3-phase PMSM using dual-vector-based PSO.

Reference^35^ introduced DRL-based MPC to boost the efficiency of EVs. Reference^36^ proposed control methodology for a 3-phase PMSM using several optimization techniques and RL. Reference^37^ suggested a RL control for a 3-phase PMSM, based on the twin delay deep deterministic (TD3) approach. Reference^38^ compared the traditional PID method and an RL to control a PMSM, employing vector control (VC).

A presentation of a hybrid adaptive nonlinear control and a deadbeat observer to enhance the efficiency of a 3-phase PMSM was given in^39^ to overcome the changes in load conditions and motor parameters. In^40^ an adaptive fuzzy proportional-integral control approach for a 3-phase PMSM was presented. An adaptive extended state observer was presented in^41^ to address peak issues and compensate for mismatched disturbances in a non-cascade-controlled PMSM in an EV. An investigation of the effect of various hybrid EV powertrains, including fuel cells, batteries, and supercapacitors, on component efficiency was given in^42^. A fractional order PID controller based on an adaptive neuro-fuzzy inference system for EV, using a DC motor was presented in^43^. A simulation model for EV-based PMSM traction applications using a model-based design approach was given in^44^. A control approach for EV-based PMSM, including the advantages of conventional proportional resonance and PI controllers, was presented in^45^. An FOC PMSM drive system was presented in^46^, employing a hysteresis current controller and a PWM-operated current controller for high-efficiency EVs. Several EV battery’s performance was analyzed in^47^ using different drive cycles. A control methodology for an EV-based PMSM was given in^48^ that employed a robust non-linear MPC in a cascaded structure, combined with SVPWM. In^49^ a control approach for a three-phase PMSM supplied by a multi-level inverter for EV applications was introduced. Reference^50^ proposed a design and energy management strategy for a solar-powered station intended for charging EVs. An energy management system incorporating a supercapacitor and two types of batteries was proposed in^51^ to enhance efficiency and extend the driving range of electric vehicles. In^52^, a DTC approach for doubly-fed induction motors is proposed, wherein a PI controller is employed. The controller parameters are optimally tuned using a Genetic Algorithm, enhancing the dynamic performance and robustness of the drive system under varying operational conditions^53^. presented an online-tuned MPCC strategy based on the adaptive neuro-fuzzy inference system (ANFIS) to improve the performance of an EV drive system employing PMSM. An ANFIS with hysteresis current control (HCC) methodology for EV based PMSM drive system was presented in^54^ to improve the system performance compared with conventional PI controller with HCC. In^55^ an ANFIS based sliding mode current controller (SMCC) for a PMSM to improve EV propulsion.

In this article, an FTC approach for an EV drive system based 5-phase IPMSM that is subjected to open-phase fault is studied. Two drive cycles are used to test the FTC approach; these drive cycles are ECE-15 and custom IM240. The 5-phase IPMSM is adopted due to its several benefits. The presented approach depends on utilizing a multi-layer perceptron (MLP) ANN to select the optimum gains of the PI controller online, i.e., online tuning. This approach is compared with the classical approach of obtaining the PI controller gains offline, i.e., offline tuning. The offline gains of the PI controller are obtained using the honey badger optimization technique. To enhance the overall system efficiency, the motor is operated at MTPA. The current harmonics and torque ripples are reduced by utilizing model predictive control to control the five-phase VSI based on a desired cost function. The validity of the proposed approach is verified by obtaining simulation results using the Matlab Simulink package.

This paper’s significant contributions include:

- A fault tolerant control approach based on MLP-ANN online tuning for a high-performance EV drive system is suggested and verified using two drive cycles.
- The presented FTC approach is compared with the recent classical metahuristic optimization technique (MHOT) called Honey Badger Optimization technique.
- Improving the torque and current profiles during the fault by utilizing the presented MPC cost function.
- A MATLAB SIMULINK is carried out for the considered EV drive systrem to validate the presented control method.

Table 1 indicates a comparison between this work and several recent published works.

**Table 1:** Several recent works and this work comparison.

Refs.	Year	Objective	Motor type	Control Method	AI online	Metaheuristic optimization technique	Drive cycle	EV application	Energy saving	Fault tolerant control	Main Outcomes
Li et al.^9^	2022	Elimination of the 3rd harmonic component.	5-phase PMSM	Sensorless control	No	No	No	No	No	No	Reducing torque ripples without needing motor parameters for all speed range of operation.
Bahar and Omar^12^	2022	Reduction of current harmonics and torque ripple.	5-phase PMSM	MP-DTC	No	No	No	No	No	No	Deliver rapid dynamic response and excellent steady-state performance.
Huang et al.^13^	2023	Eliminate the impact of motor parameters variation on the drive system performance.	5-phase PMSM	MPCC	No	No	No	No	No	No	Improvement of steady-state performance of the drive system.
Rajanikanth et al.^14^	2024	Decreasing computational time relative to traditional MPC	5-phase PMSM	MPCC	No	No	No	No	No	No	Improved steady-state torque and flux responses.
Bensalem et al.^19^	2022	Active FTC for an EV speed tracking with sensor failure.	5-phase PMSM	Extended Kalman filter observer and backstepping controllers	No	No	Yes	Yes	No	Yes	Improved fault detection and speed tracking response
Du et al.^22^	2023	FTC based MPC method to reduce torque ripple of 5-phase PMSM drive under single and multiple open-circuit faults (OCFs).	5-phase PMSM	Model predictive current control	No	No	No	No	No	Yes	A general reference current generation method based on optimal current control is developed to suppress torque ripple.
Xu et al.^23^	2024	To enhance output torque and improve FTC for a 5-ph IPMSM used in aerospace application.	5-phase PMSM	Quasi-proportional resonant control	No	Yes	No	No	No	Yes	Reduced torque fluctuation due to the reluctance torque during fault.
Li et al.^24^	2024	Study the FTC based on MPC was for a 5-phase PMS hub motor under OPF and two OPFs	5-phase PMS hub motor	Model predictive current control	No	No	No	No	No	Yes	Improved fault-tolerant operation capability of 5-phase PMS hub motor
Zeghlache et al.^25^	2024	Introducing a robust FTC for a 5-ph PMSM affected by the third harmonic under an OPF.	5-phase PMSM	FOC & SMC	No	No	No	No	No	Yes	Reduced torque ripples and speed fluctuations under fault.
Zhou et al.^26^	2024	Presenting an FTC method for a 5-phase PMSM under OPF based an indirect correction approach of imaginary vectors	5-phase PMSM	DTC	No	No	No	No	No	Yes	Good dynamic and static responses.
Sangar et al.^53^	2024	Improving the performance of PMSM in EV drive system using online-tuned MPCC algorithm based on ANFIS	3-phase PMSM	MPCC based ANFIS	Yes	No	Yes	Yes	No	No	Improved the dynamic speed response and reduced torque ripples of EV drive system based PMSM
Sangar et al.^54^	2025	Enhancing the performance of EV based PMSM drive system using ANFIS with HCC compared with conventional PI controller with HCC.	3-phase PMSM	ANFIS with HCC	Yes	No	Yes	Yes	No	No	Improved performance of EV drive system based PMSM
Sangar et al.^55^	2025	Improving the performance of EV based PMSM drive system using ANFIS based SMCC	3-phase PMSM	ANFIS based SMCC	Yes	No	Yes	Yes	No	No	Enhanced EV repulsion
This Work		Improving Speed tracking control and energy saving for an EV based IPMSM drive system under OPF using MLP-ANN that is tested by two drive cycles.	5-phase PMSM	MPC based MLP-ANN online tuning	Yes	yes	Yes	Yes	Yes	Yes	The response of EV drive system under OPF is superior than offline PI tuning. In addition to this energy saving is attained.

The remaining sections are structured as follows: Sect. EV drive system modeling under OPF explores the EV drive system modeling under OPF. Section MPC strategy presents the suggested MPC. Section Proposed adaptive MLP-ANN  explains the MLP-ANN online tuning. Section Results presents the results of the study. Section Conclusion introduces the conclusion.

## EV drive system modeling under OPF

**Fig. 1:**
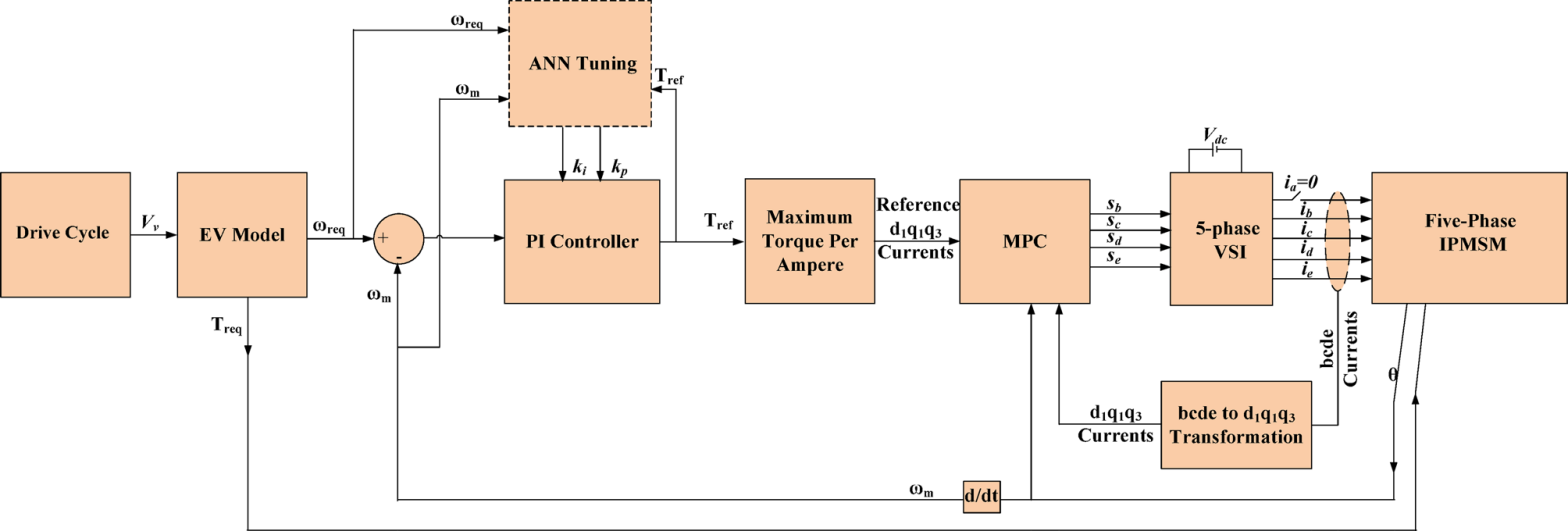
Adopted EV drive system.

Figure 1 shows the electric vehicle drive system under consideration. It consists of several subsystems. In the following subsections, the mathematical model of each subsystem of the EVDS will be introduced.

### Drive cycle model

**Fig. 2:**
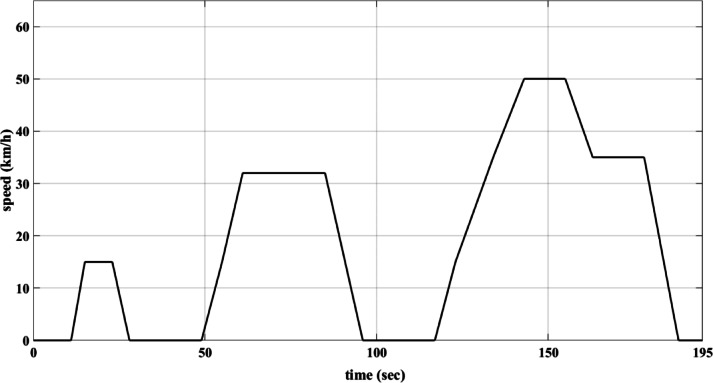
ECE-15 drive cycle.

**Fig. 3:**
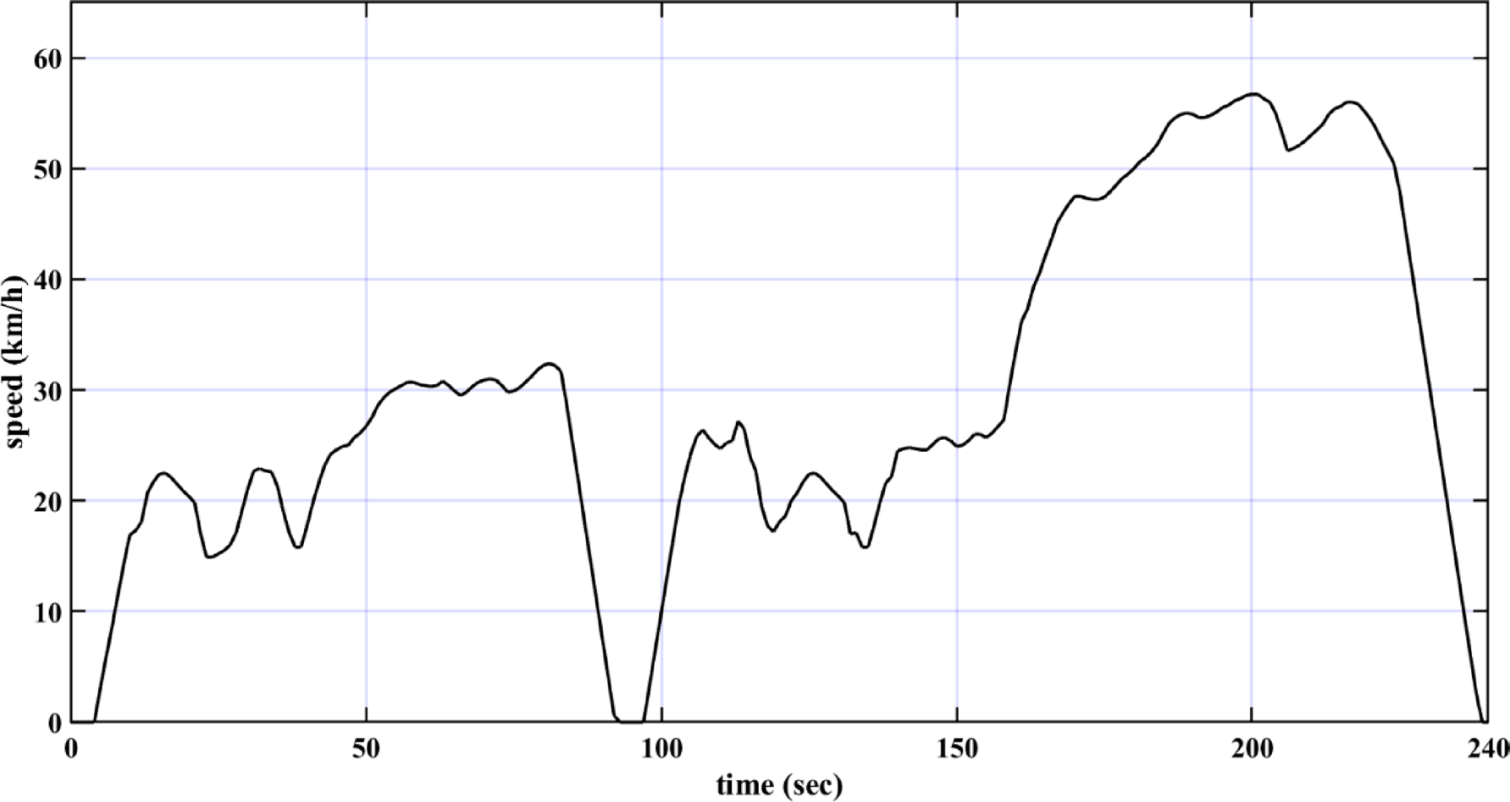
IM240 drive cycle.

The drive cycles are represented through their speed-time graphs. This study adopts the European drive cycle (ECE-15), depicted in Fig. 2^42^, and the customized inspection and maintenance drive cycle (IM240), shown in Fig. 3^56^. Table 2 outlines the characteristics of these two drive cycles. These drive cycles are adopted because they are designed to represent real-world driving conditions, including acceleration, deceleration, and cruising. This helps in evaluating the proposed method of control of the EV drive system under typical usage scenarios. To ensure the validity of the proposed method of control, the two different drive cycles are used instead of only one.

**Table 2:** Characteristics of the drive cycles.

Features	ECE-15	Custom IM240
Distance	0.9941 km	3.1 km
Total time	195 s	240 s
Average speed (incl. stops)	18.35 km/h	29.4 km/h
Maximum speed	50 km/h	57.6 km/h

### EV model

**Fig. 4:**
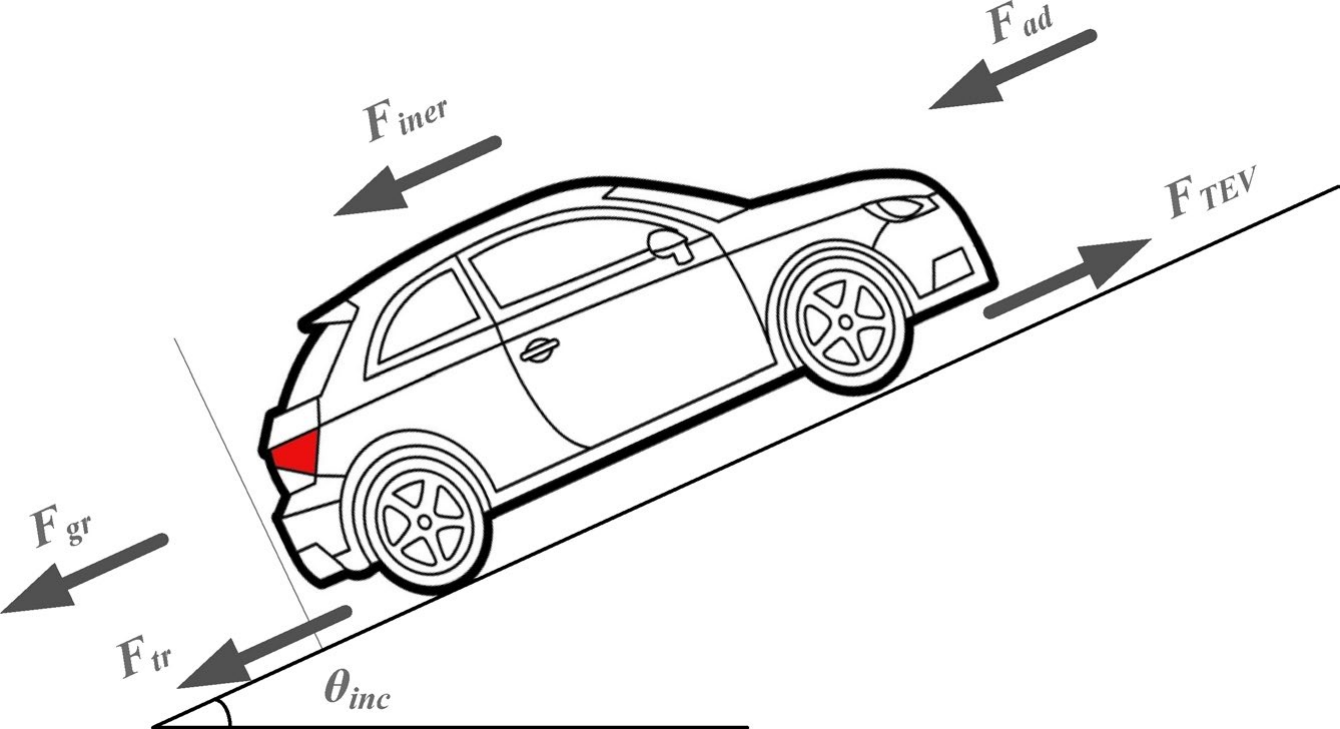
Forces affecting an electric vehicle.

The velocity from the drive cycle serves as the input for the vehicle model. The outputs of the model include the required vehicle torque and motor speed. The vehicle torque is determined by calculating the total force, which is the sum of all forces acting on the vehicle. These forces include aerodynamic force, rolling resistance, gradient force, and inertia force, as illustrated in Fig. 4.

The aerodynamic force, *F*_*ad*_, can be formulated as follows^2,44,49^:

1 $$F_{{ad}} = \frac{1}{2}\rho _{a} C_{{ad}} A_{{fa}} (V_{{EV}} + V_{w} )^{2}$$

where *ρ*_*a*_ is the air density in kg/m^3^, *C*_*ad*_ is the coefficient of aerodynamic drag, *A*_*fa*_ is the frontal area of the EV in m^2^, *V*_*EV*_ is the EV velocity in m/s and *V*_*w*_ is the speed of wind in m/s. The tyre rolling force, *F*_*tr*_, can be expressed as follows^2,44,49^:

2 $$F_{{tr}} = m_{{EV}} g_{{acc}} C_{{tr}} \cos \theta _{{inc}}$$

where*m*_*EV*_ is the EV mass in kg, g_acc_ is the acceleration of gravity, *C*_*tr*_ is the coefficient of tyre rolling resistance and *θ*_*inc*_ is the EV inclination angle in degrees. The gradient force, *F*_*gr*_, is represented by the following formula^2,44,49^:

3 $$F_{{gr}} = m_{{EV}} g_{a} \sin \theta _{{inc}}$$

The inertia force, *F*_*iner*_, can be formulated as follows^2,44,49^:

4 $$F_{{iner}} = m_{{EV}} a_{{EV}}$$

where *a*_*EV*_ is the EV acceleration. Thus, the total EV force can be derived using the following formula^2,44,49^:

5 $$F_{{TEV}} = F_{{ad}} + F_{{tr}} + F_{{gr}} + F_{{iner}}$$

As a result, the required motor torque can be derived from^2,44,49^:

6 $$T_{T} = F_{{TEV}} R_{w} /G_{{ratio}}$$

where *R*_*w*_ is the EV wheel radius and *G*_*ratio*_ is the gear ratio. Additionally, the required motor speed can be derived from^2,44,49^:

7 $$\omega _{{mreq}} = G_{{ratio}} V_{{EV}} /R_{w}$$

Therefore, the required power for the electric vehicle can be determined from:

8 $$P_{{EV}} = T_{T} \omega _{{mreq}}$$

Furthermore, the required energy can be expressed as follows:

9 $$E_{{EV}} = \int {P_{{EV}} dt}$$

### Five-phase VSI model under OPF

Since the system is studied under OPF, Phase-A is assumed to be opened. Consequently, the VSI can be modeled in terms of the voltages (*v*_*b*_ to *v*_*e*_), that are dependent on the switching functions of the VSI^57^.

10 $$\left[ {\begin{array}{*{20}c} {v_{b} } \\ {v_{c} } \\ {v_{d} } \\ {v_{e} } \\ \end{array} } \right] = \frac{{V_{{dc}} }}{4}\left[ {\begin{array}{*{20}c} 3 & { - 1} & { - 1} & { - 1} \\ { - 1} & 3 & { - 1} & { - 1} \\ { - 1} & { - 1} & 3 & { - 1} \\ { - 1} & { - 1} & { - 1} & 3 \\ \end{array} } \right].\left[ {\begin{array}{*{20}c} {s_{b} } \\ {s_{c} } \\ {s_{d} } \\ {s_{e} } \\ \end{array} } \right]$$

In Eq. (10), V_dc_ denotes the voltage of the battery. The switching functions, S_b_ to S_e_, illustrate the various VSI switching states. A switching function assumes a value of one when the upper switch in a leg is ON, and zero when the lower switch in that leg is OFF. There are 16 switching states in the open phase fault case. The MPC determines the optimal switching state that results in a minimum cost function. Based on the selected state from the 16 feasible options, gate pulses are delivered to the ten switches of the Voltage Source Inverter (VSI).

### 5-phase IPMSM model under OPF

The 5-phase IPMSM is expressed in terms of the dq model in the synchronously rotating reference frame. The details of this model were given in reference^58^. The post-fault current reconfiguration strategy is founded on maintaining a constant and unaltered permanent magnet flux linkage, along with its corresponding back electromotive force, within the synchronously rotating reference frame. The Clark and Park transformations are modified for the case of open phase fault to result in the resultant flux space vector follows a circular trajectory. Also, in the Clarke transformation matrix, the third row, associated with the direct third harmonic component, is excluded due to its lack of orthogonality with the remaining rows, which are mutually orthogonal. Additionally, the first column, representing the phase ‘a’ component, is eliminated since its contribution becomes null under fault conditions^58^. The modified Clark and Park matrices transformations in reference^58^, are used to obtain the transformations from voltage BCDE to DQ as follows:

11 $$\left[ {\begin{array}{*{20}c} {v_{{d1}} } \\ {v_{{q1}} } \\ {v_{{q3}} } \\ {v_{0} } \\ \end{array} } \right] = \frac{2}{5}\left[ {\begin{array}{*{20}c} {\cos (\alpha - \theta ) - \cos (\theta )} & {\cos (2\alpha - \theta ) - \cos (\theta )} & {\cos (2\alpha + \theta ) - \cos (\theta )} & {\cos (\alpha + \theta ) - \cos (\theta )} \\ {\sin (\alpha - \theta ) + \sin (\theta )} & {\sin (2\alpha - \theta ) + \sin (\theta )} & { - \sin (2\alpha + \theta ) + \sin (\theta )} & { - \sin (\alpha + \theta ) + \sin (\theta )} \\ { - \sin (2\alpha )} & {\sin \alpha } & { - \sin \alpha } & {\sin (2\alpha )} \\ 1 & 1 & 1 & 1 \\ \end{array} } \right].\left[ {\begin{array}{*{20}c} {v_{b} } \\ {v_{c} } \\ {v_{d} } \\ {v_{e} } \\ \end{array} } \right]$$

where the value of α is 2π/5, *v*_*d1*_ and *v*_*q1*_ are the DQ fundamental stator voltages, *v*_*q3*_ is the quadrature 3rd harmonic voltage, and θ is the rotor position angle.

To transform from the DQ to the BCDE quantities, a matrix inversion using MATLAB symbolic toolbox is applied to Eq. (11) to obtain the following DQ to the BCDE equation:

12 $$\left[ {\begin{array}{*{20}c} {i_{{bs}} } \\ {i_{{cs}} } \\ {i_{{ds}} } \\ {i_{{es}} } \\ \end{array} } \right] = \left[ {\begin{array}{*{20}c} {\sin \alpha .\sin \theta - \frac{{1.25\cos \theta }}{{\cos (2\alpha ) - \cos \alpha }}} & {\sin \alpha .\cos \theta + \frac{{1.25\sin \theta }}{{\cos (2\alpha ) - \cos \alpha }}} & { - \sin (2\alpha )} & {\frac{{1.25\cos (2\alpha - 1)}}{{\cos (2\alpha ) - \cos \alpha }}} \\ {\sin (2\alpha ).\sin \theta + \frac{{1.25\cos \theta }}{{\cos (2\alpha ) - \cos \alpha }}} & {\sin (2\alpha ).\sin \theta - \frac{{1.25\sin \theta }}{{\cos (2\alpha ) - \cos \alpha }}} & {\sin \alpha } & { - \frac{{1.25\cos (\alpha - 1)}}{{\cos (2\alpha ) - \cos \alpha }}} \\ { - \sin (2\alpha ).\sin \theta + \frac{{1.25\cos \theta }}{{\cos (2\alpha ) - \cos \alpha }}} & { - \sin (2\alpha ).\cos \theta - \frac{{1.25\sin \theta }}{{\cos (2\alpha ) - \cos \alpha }}} & { - \sin \alpha } & { - \frac{{1.25\cos (\alpha - 1)}}{{\cos (2\alpha ) - \cos \alpha }}} \\ { - \sin \alpha .\sin \theta - \frac{{1.25\cos \theta }}{{\cos (2\alpha ) - \cos \alpha }}} & { - \sin \alpha .\cos \theta + \frac{{1.25\sin \theta }}{{\cos (2\alpha ) - \cos \alpha }}} & {\sin (2\alpha )} & {\frac{{1.25\cos (2\alpha - 1)}}{{\cos (2\alpha ) - \cos \alpha }}} \\ \end{array} } \right].\left[ {\begin{array}{*{20}c} {i_{{d1}} } \\ {i_{{q1}} } \\ {i_{{q3}} } \\ {i_{0} } \\ \end{array} } \right]$$

where *i*_*d1*_ and *i*_*q1*_ are the DQ fundamental stator currents, and *i*_*q3*_ is the quadrature 3rd harmonic stator currents.

The voltage differential equation of the 5-phase IPMSM in case of open-phase fault is explained in [58] and can be expressed as follows:

13 $$\left[ {\begin{array}{*{20}c} {v_{{d1}} } \\ {v_{{q1}} } \\ {v_{{q3}} } \\ \end{array} } \right] = \left[ {\begin{array}{*{20}c} {R_{s} } & 0 & 0 \\ 0 & {R_{s} } & 0 \\ 0 & 0 & {R_{s} } \\ \end{array} } \right].\left[ {\begin{array}{*{20}c} {i_{{d1}} } \\ {i_{{q1}} } \\ {i_{{q3}} } \\ \end{array} } \right] + \left[ {\begin{array}{*{20}c} {\frac{{d\lambda _{{d1}} }}{{dt}}} \\ {\frac{{d\lambda _{{q1}} }}{{dt}}} \\ {\frac{{d\lambda _{{q3}} }}{{dt}}} \\ \end{array} } \right] - \omega \left[ {\begin{array}{*{20}c} 0 & 1 & 0 \\ { - 1} & 0 & 0 \\ 0 & 0 & { - 3} \\ \end{array} } \right].\left[ {\begin{array}{*{20}c} {\lambda _{{d1}} } \\ {\lambda _{{q1}} } \\ {\lambda _{{d3}} } \\ \end{array} } \right]$$

where:

14 $$\begin{gathered} \lambda _{{d1}} = L_{{d1}} i_{{d1}} + \lambda _{{1m}} \hfill \\ \lambda _{{q1}} = L_{{q1}} i_{{q1}} + L_{{m13}} i_{{q3}} \hfill \\ \lambda _{{d3}} = L_{{m13}} i_{{d1}} + \lambda _{{3m}} \hfill \\ \lambda _{{q3}} = L_{{q3}} i_{{q3}} + L_{{m13}} i_{{q1}} \hfill \\ \end{gathered}$$

and *R*_*s*_ is the winding resistance, ω is the angular speed, *L*_*d1*_ and *L*_*q1*_ represent the DQ fundamental inductances, *L*_*d3*_ and *L*_*q3*_ represent the DQ 3rd harmonic inductances, *L*_*m13*_ is the mutual inductance, and *λ*_*1m*_, *λ*_*3m*_ are the PM fundamental and 3rd component fluxes.

The motor differential equation, Eq. (13), can be rewritten using MATLAB symbolic toolbox in the following form:

15 $$D[I] = [LL][V] - [LR].[I] + \omega [LG].[I] - \omega [L\lambda ]$$

where D is the operator d/dt, $$[I] = [\begin{array}{*{20}c} {i_{{d1}} } & {i_{{q1}} } & {i_{{q3}} } \\ \end{array} ]^{T}$$, $$[V] = [\begin{array}{*{20}c} {v_{{d1}} } & {v_{{q1}} } & {v_{{q3}} ]} \\ \end{array} ^{T},$$

$$[LL] = \left[ {\begin{array}{*{20}c} {\frac{1}{{L_{{d1}} }}} & 0 & 0 \\ 0 & {\frac{{ - L_{{q3}} }}{{L_{{m13}}^{2} - L_{{q1}} L_{{q3}} }}} & {\frac{{L_{{m13}} }}{{L_{{m13}}^{2} - L_{{q1}} L_{{q3}} }}} \\ 0 & {\frac{{L_{{m13}} }}{{L_{{m13}}^{2} - L_{{q1}} L_{{q3}} }}} & {\frac{{ - L_{{q1}} }}{{L_{{m13}}^{2} - L_{{q1}} L_{{q3}} }}} \\ \end{array} } \right]$$

$$[LR] = \left[ {\begin{array}{*{20}c} {\frac{{R_{s} }}{{L_{{d1}} }}} & 0 & 0 \\ 0 & {\frac{{ - L_{{q3}} R_{s} }}{{L_{{m13}}^{2} - L_{{q1}} L_{{q3}} }}} & {\frac{{L_{{m13}} R_{s} }}{{L_{{m13}}^{2} - L_{{q1}} L_{{q3}} }}} \\ 0 & {\frac{{L_{{m13}} R_{s} }}{{L_{{m13}}^{2} - L_{{q1}} L_{{q3}} }}} & {\frac{{ - L_{{q1}} R_{s} }}{{L_{{m13}}^{2} - L_{{q1}} L_{{q3}} }}} \\ \end{array} } \right]$$

$$[LG] = \left[ {\begin{array}{*{20}c} 0 & {\frac{{L_{{q1}} }}{{L_{{d1}} }}} & {\frac{{L_{{m13}} }}{{L_{{d1}} }}} \\ {\frac{{L_{{d1}} L_{{q3}} - 3L_{{m13}}^{2} }}{{L_{{m13}}^{2} - L_{{q1}} L_{{q3}} }}} & 0 & 0 \\ {\frac{{L_{{m13}} (3L_{{q1}} - L_{{d1}} )}}{{L_{{m13}}^{2} - L_{{q1}} L_{{q3}} }}} & 0 & 0 \\ \end{array} } \right]$$

$$[L\lambda ] = \left[ {\begin{array}{*{20}c} 0 \\ {\frac{{3\lambda _{{3m}} L_{{m13}} - \lambda _{{1m}} L_{{q3}} }}{{L_{{m13}}^{2} - L_{{q1}} L_{{q3}} }}} \\ {\frac{{\lambda _{{1m}} L_{{m13}} - 3\lambda _{{3m}} L_{{q1}} }}{{L_{{m13}}^{2} - L_{{q1}} L_{{q3}} }}} \\ \end{array} } \right]$$

The developed torque can be obtained with *i*_*d3*_=0 as follows:

16 $$T_{e} = \frac{5}{2}\frac{p}{2}[\lambda _{{d1}} i_{{q1}} - \lambda _{{q1}} i_{{d1}} + 3\lambda _{{d3}} i_{{q3}} ]$$

where p is the number of poles.

Substitution of Eq. (14) into Eq. (16) gives:

17 $$T_{e} = \frac{5}{2}\frac{p}{2}[(L_{{d1}} - L_{{q1}} )i_{{d1}} i_{{q1}} + 2L_{{m13}} i_{{d1}} i_{{q3}} + \lambda _{{1m}} i_{{q1}} + 3\lambda _{{3m}} i_{{q3}} ]$$

The formula of the mechanical equation may be written as^59^:

18 $$D\omega _{m} = \frac{{T_{e} - T_{l} (\omega _{m} )}}{J}$$

where J is the inertia, and $T_{l} (\omega _{m} )$is given by:

19 $$T_{l} (\omega _{m} ) = T_{L} + T_{{fw}}$$

where $T_{L}$the load is torque and $T_{{fw}}$ is the friction and windage torque.

### MTPA operating mode model

For maximum efficiency, the 5-phase IPMSM operates at MTPA. When neglecting L_m13_, the fundamental and 3rd -harmonic DQ currents achieving MTPA can be derived from^59^:

20 $$i_{{d1}} = \frac{{\lambda _{{1m}} }}{{2(L_{{q1}} - L_{{d1}} )}} - \sqrt {\frac{{\lambda _{{1m}}^{2} }}{{4(L_{{q1}} - L_{{d1}} )^{2} }} + i_{{q1}}^{2} }$$

21 $$i_{{q1}} = \frac{{T_{{e1}} }}{{\frac{5}{2}\frac{p}{2}[(L_{{d1}} - L_{{q1}} )i_{{d1}} + \lambda _{{1m}} ]}}$$

Also, the 3rd harmonic quadrature current can be obtained from^59,60^:

22 $$i_{{q3}} = k\sqrt {i_{{d1}}^{2} + i_{{q1}}^{2} } \cos \{ 3[\tan ^{{ - 1}} (\frac{{i_{{d1}} }}{{i_{{q1}} }})]\}$$

## MPC strategy

Model Predictive Control (MPC) consists of a plant model and an optimizer. Its primary objective is to choose the optimal set of inputs for the system by predicting its future behavior. The plant model generates forecasts based on past states to anticipate the next ones. At each sampling period, the optimizer uses the predicted states and the desired trajectory to solve the optimization problem over the forecasting horizon, thereby determining the optimal set of inputs for future operations^59,61^.

To effectively implement Model Predictive Control, it is crucial to discretize the 5-phase Interior Permanent Magnet Synchronous Motor model. Thus, Eq. (15) is converted into its discrete form as in the following formula:

23 $$[I(k + 1)] = [I(k)] + T_{s} \{ LL][V] - [LR].[I(k)] + \omega [LG].[I(k)] - \omega [L\lambda ]\}$$

where T_s_ is the sampling period, [I(k)] is the current value dq-current matrix [I], Eq. (15), and [I(k + 1)] is the predicted value of the dq-current matrix [I].

The main objective of the MPC is to minimize torque error. The torque error can be minimized by minimizing the DQ current errors. Consequently, the cost function (CF) that minimizes the DQ current errors can be written in the following form^59^:

The primary goal of Model Predictive Control is to reduce torque error. This can be achieved by minimizing the errors in the DQ currents. Therefore, the cost function (CF) that reduces the DQ current errors can be expressed as follows:

24 $$C.F = [i_{{d1r}} - i_{{d1}} (k + 1)]^{2} + [i_{{q1r}} - i_{{q1}} (k + 1)]^{2} + [i_{{q3r}} - i_{{q3}} (k + 1)]^{2}$$

Here, (*i*_*d1r*_, *i*_*q1r*_, and *i*_*q3r*_) denote the reference DQ currents. They can be calculated with the aid of Eqs. (20), (21), and (22). The optimal 5-phase VSI switching functions are chosen from the 16 possible switching states in the case of open-phase fault based on the lowest cost function.

## Proposed adaptive MLP-ANN

The mathematical model of a classic PI controller can be described as follows:

25 $$\:u\left(t\right)=\:{K}_{P}{e}_{t}\left(t\right)+{\:K}_{i}\int\:{e}_{t}\left(t\right)\:dt$$

Here, $\:{K}_{P}$, $\:{\:K}_{i}$ and $\:{e}_{t}\left(t\right)$are the proportional and integral gains and error signals, respectively. $\:u\left(t\right)$ is the controller output. These PI parameters are set to fixed values using techniques such as the Ziegler-Nichols first tuning method^62^.

**Fig. 5:**
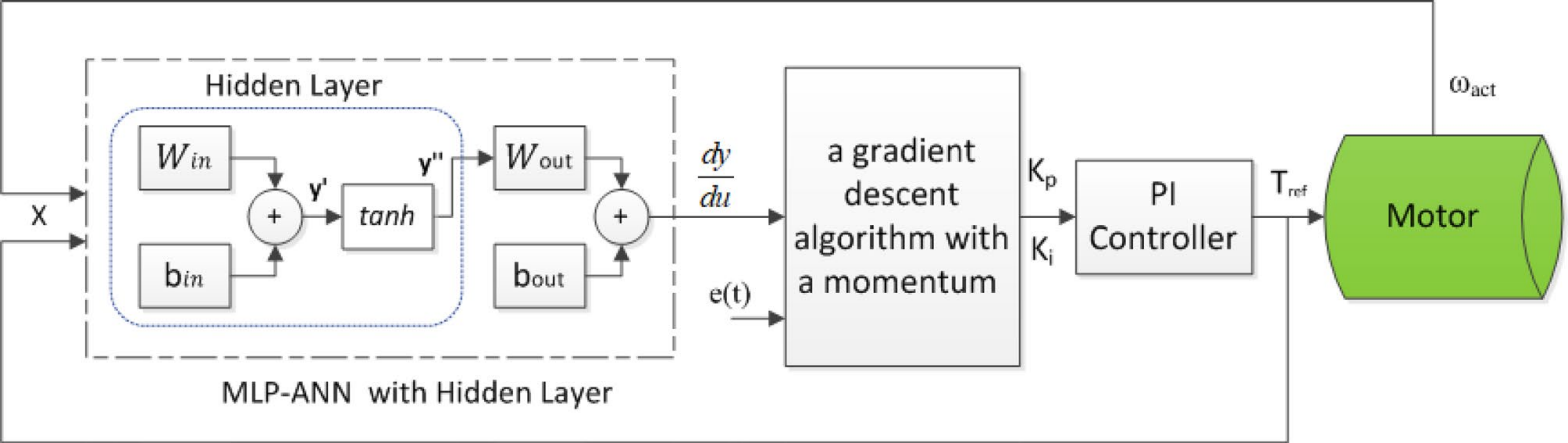
The Proposed Adaptive PI-ANN Controller.

Artificial Neural Networks (ANNs) offer a data-driven method for modeling system behavior by utilizing learning mechanisms based on error minimization criteria. In this research, a multi-layer perceptron (MLP) ANN was employed for offline training. The MLP architecture includes a single hidden layer with 20 neurons, as illustrated in Fig. 5. The activation function used is Tanh (Hyperbolic Tangent), and the model is trained using the Stochastic Gradient Descent optimizer with a learning rate of 0.9. Training was conducted over 150 iterations. The structure of the MLP-ANN and its corresponding learning error criteria are depicted as follows^62^:

26 $$\:\widehat{y}={W}_{out}^{T}\text{tanh}\left({W}_{in}^{T}X+{b}_{in}\right)+{b}_{out}$$

27 $$\:E=\frac{1}{2}\:\sum\:{\left(e\right)}^{2}=\frac{1}{2}\:\sum\:{(y-\widehat{y})}^{2}$$

Where $\:X$ is the input vector, $\:y$ is the actual output, $\:\widehat{y}$ is the predicted output, $\:e$ is the error signal, and $\:E$ denotes MSE used as the CF. Where *X* represents the input vector, y denotes the actual output, $\:\widehat{y}$ is the predicted output, *e* is the error signal, and *E* stands for the Mean Squared Error (MSE) used as the cost function. The terms $\:{W}_{in}$, $\:{W}_{out}$, $\:{b}_{in}$, and $\:{b}_{out}$ represent the weights and biases of the ANN.

The presented adaptive PI-ANN control strategy leverages the MLP-ANN model^62^. By utilizing the Jacobian matrix of the system, the $\:{K}_{P}$ and $\:{\:K}_{i}$ are updated in each iteration of the control loop. The block diagram of the suggested control algorithm is depicted in Fig. 5. This approach incorporates the Jacobian matrix to compute the gradient vector, enabling the adjustment of the PI parameters using the chain rule of the backpropagation algorithm, as shown below:

28 $$\:\frac{dy}{du}\cong\:\frac{d\widehat{y}}{du}={W}_{in}^{T}{W}_{out}^{T}\frac{d{y}^{{\prime\:}{\prime\:}}}{d{y}^{{\prime\:}}}$$

29 $$\:\frac{dE}{d{K}_{P,i}}=\frac{dE}{de}\frac{de}{d\widehat{y}}\frac{d\widehat{y}}{dX}\frac{dX}{du}\frac{du}{d{K}_{P,i}}$$

Here $\:\frac{dy}{du}\cong\:\frac{d\widehat{y}}{du}$ represents the Jacobian matrix of the ANN, $\:\frac{d{y}^{{\prime\:}{\prime\:}}}{d{y}^{{\prime\:}}}=1-{tanch}^{2}\left({y}^{{\prime\:}}\right)$ is the differentiation of the function of activation, $\:\frac{dE}{d{K}_{P,i}}$ are the derivatives of the CF w.r.t the PI parameters, $\:\frac{dE}{de}$ is the derivative of the CF w.r.t the error, $\:\frac{de}{d\widehat{y}}$ is determined as -1, $\:\frac{du}{d{K}_{P,i}}$ are the derivatives of the control signal $\:u\left(t\right)$ with respect to the PI parameters, $\:\frac{du}{d{K}_{P}}={e}_{t}\left(t\right)$ and $\:\frac{du}{d{K}_{i}}={\int\:}_{0}^{\infty\:}{e}_{t}\left(t\right)\:\text{d}\text{t}$, and $\:{e}_{t}\left(t\right)$ is the closed-loop error signal.

To adaptively update the PI parameters, a gradient descent algorithm with a momentum term^62^ is employed. This iterative update rule is given as follows:

30 $$\:{K}_{P,i}\left(n\right)={K}_{P,i}\left(n-1\right)-\alpha\:V{K}_{P,i}\left(n\right)$$

31 $$\:V{K}_{P,i}\left(n\right)=\beta\:{\nabla\:}_{{K}_{P,i}}\left(n-1\right)+(1-\beta\:){\nabla\:}_{{K}_{P,i}}\left(n\right)$$

**Fig. 6:**
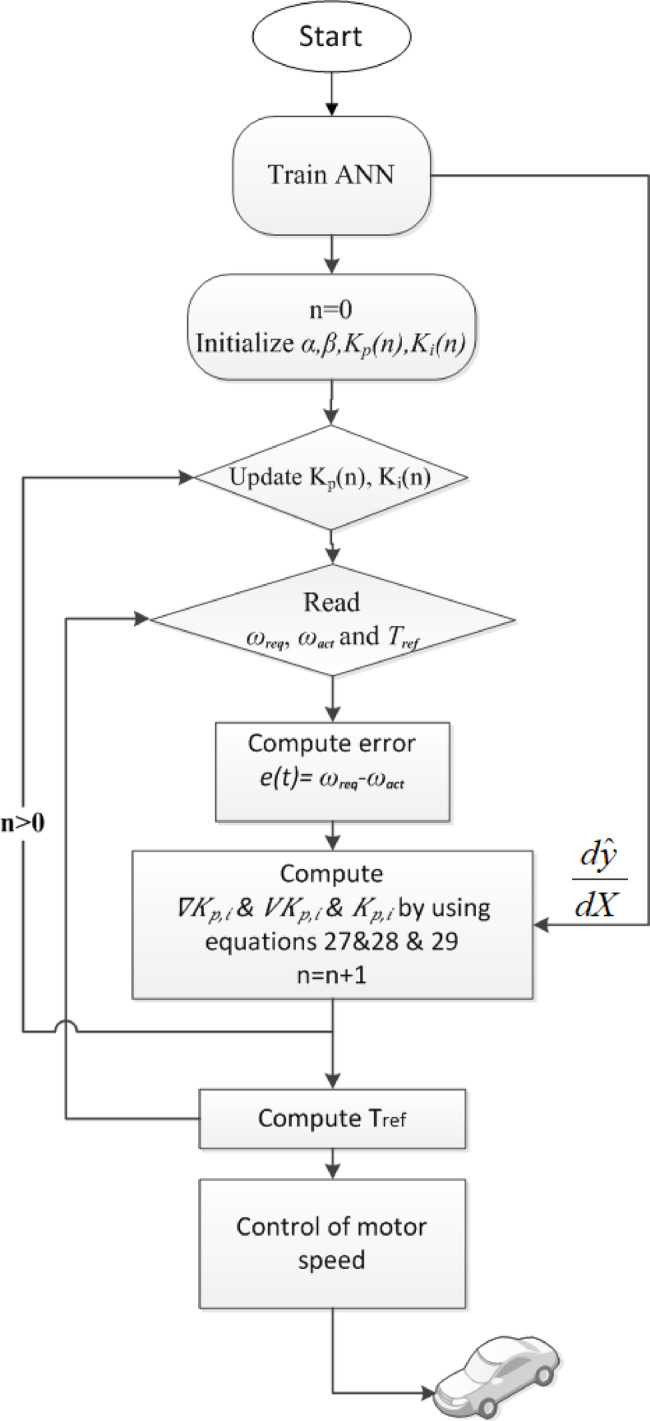
Flowchart Proposed Adaptive PI Control Algorithm.

In this equation, $\:{K}_{P,i}$ are the PI parameters, $\:{\nabla\:}_{{K}_{P,i}}=\frac{du}{d{K}_{P,i}}$ is the gradient, $\:n$ is the iteration number, $\:\alpha\:$ is the learning rate, and $\:\beta\:$ is the momentum coefficient. The flowchart for the suggested algorithm is shown in Fig. 6.

## Results

Simulation results are given for the adopted EV drive system for the ECE-15 and custom IM240 drive cycles. The results are obtained using MATLAB SIMULINK whose solver is configured to be automatic with variable step sampling time. Regarding the inverter parameters, the IGBT/Diode used as a switch with 0.001 Ω internal resistance, 100 k Ω snubber resistance. It should be noticed that the motor is operated under OPF during the whole simulation time. The EV parameters are stated in Table 3^44^. The motor data are given in Table 4^10^.

**Fig. 7:**
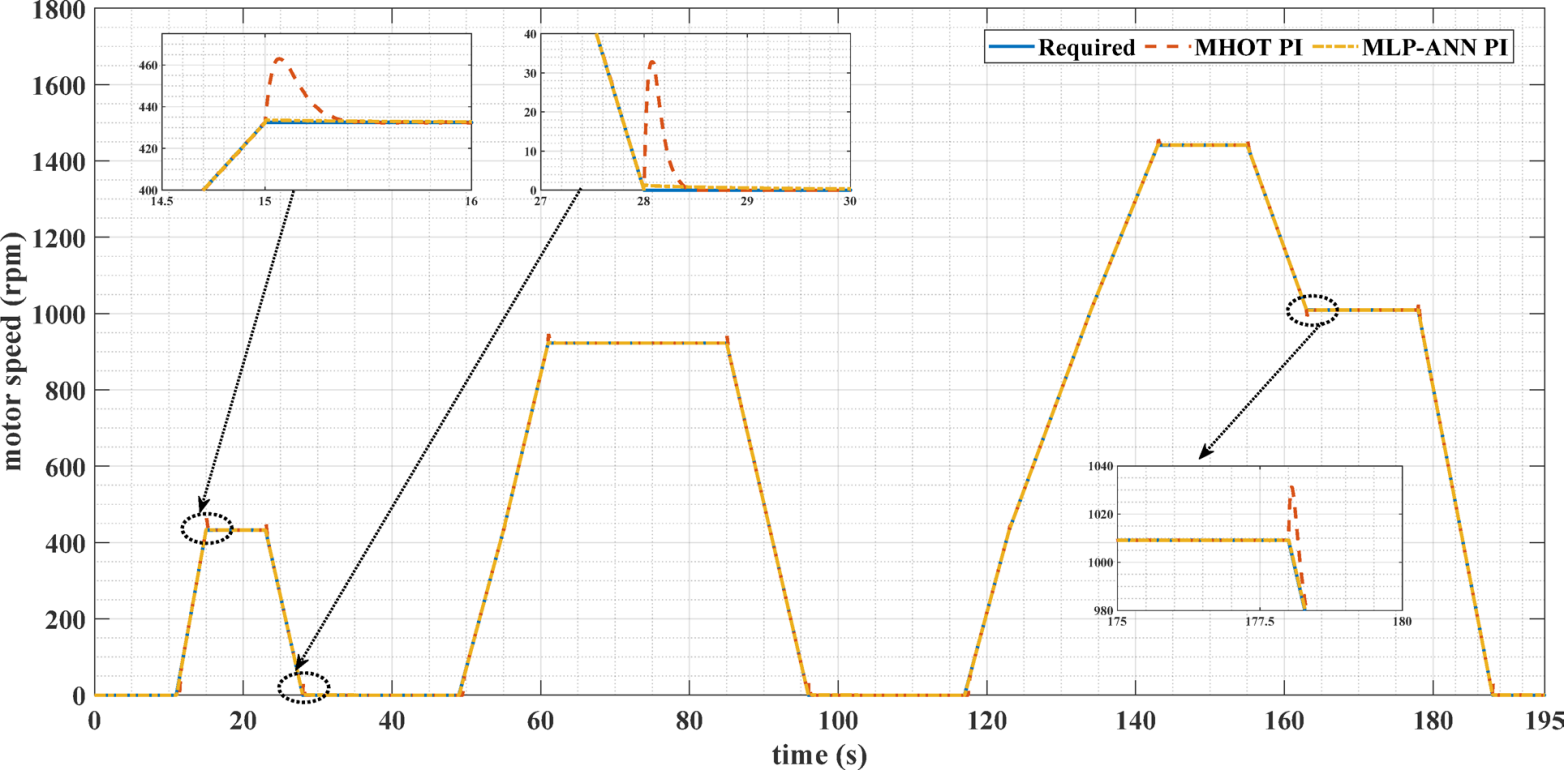
The required and measured motor speeds for the ECE-15 drive cycle when classic and online PI tuning are used.

**Fig. 8:**
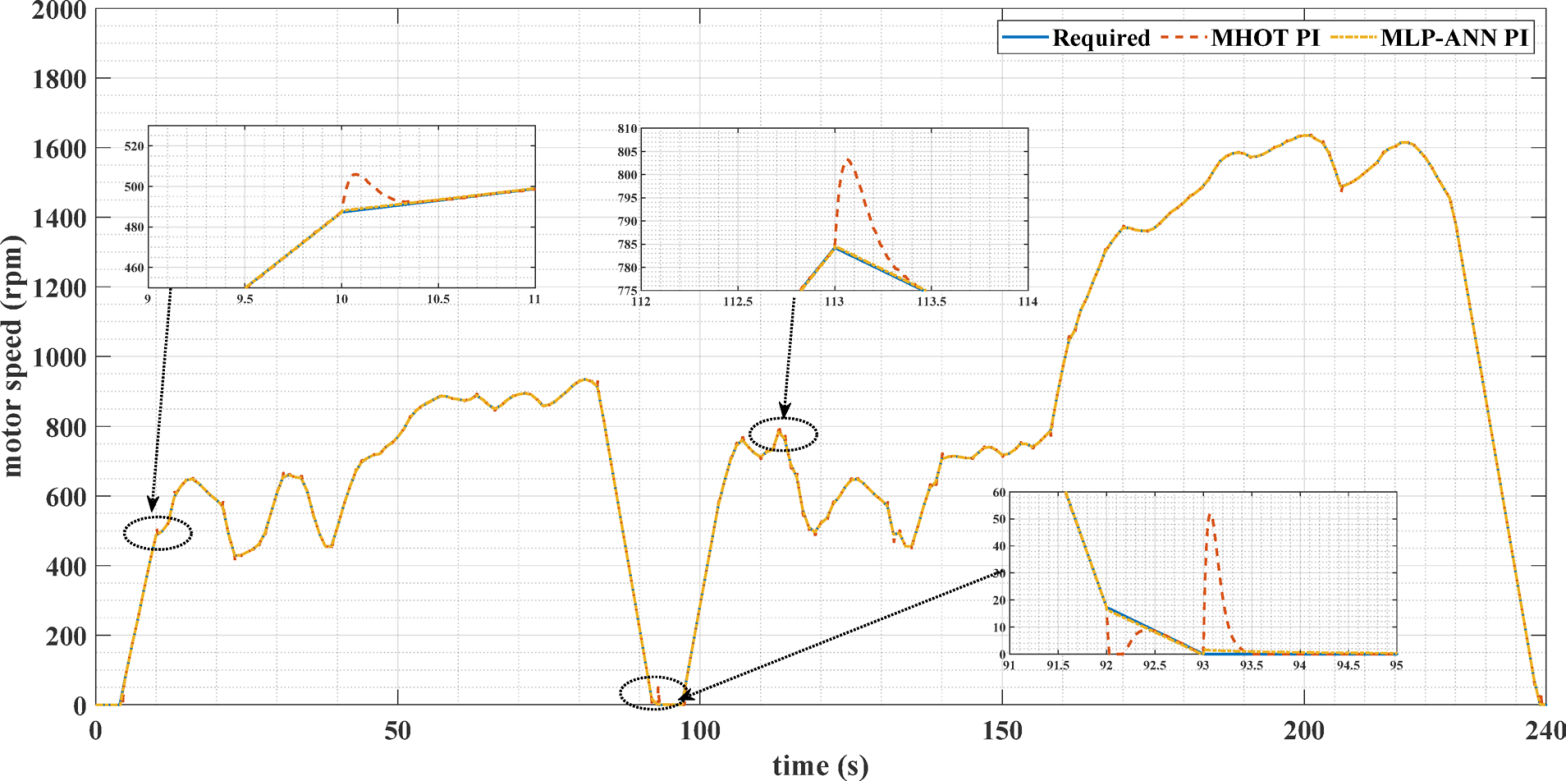
The required and measured motor speeds for the custom IM240 drive cycle, as well as online PI tuning, are utilized.

**Fig. 9:**
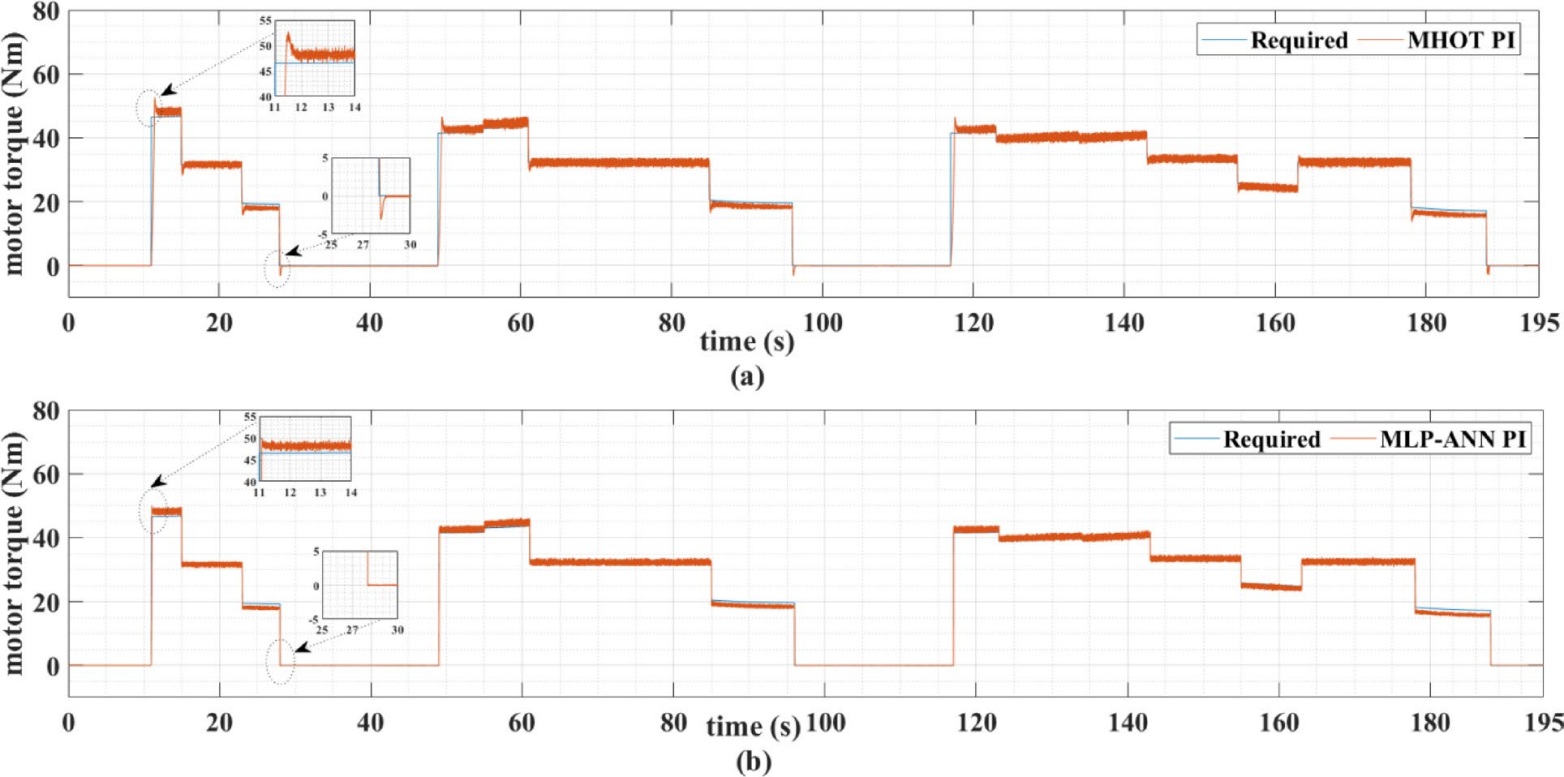
The required and actual PMSM torque of the motor for the ECE-15 drive cycles when (a) classic and (b) online PI are used.

**Fig. 10:**
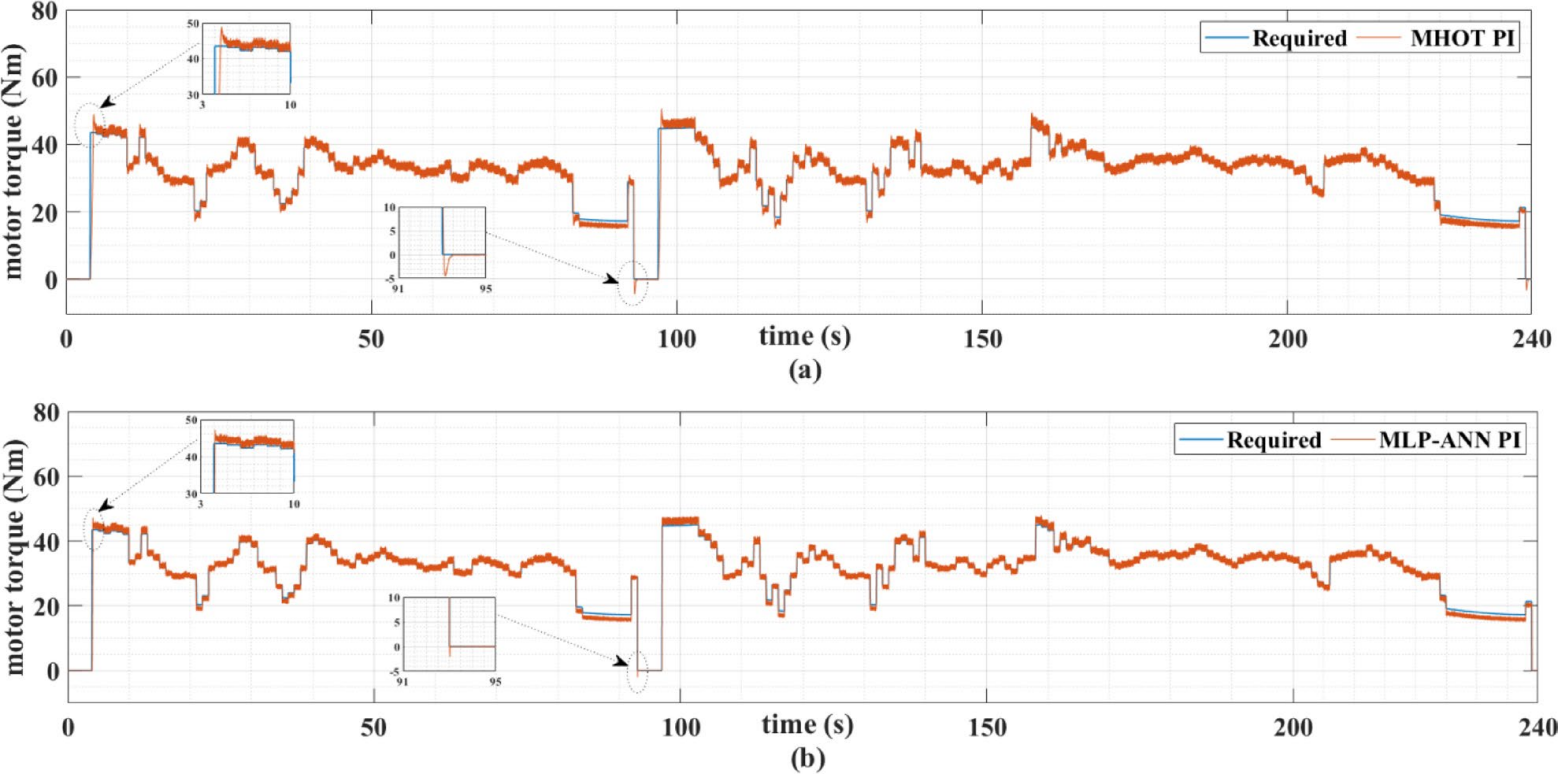
The required and actual PMSM torque for the custom IM240 drive cycle when (a) classic and (b) online PI tuning is used.

Figures 7 and 8 show the reference and measured motor speeds for both drive cycles when the classic (MHOT) and the online (MLP-ANN) tuned PI controllers are used. It can be noticed that there are several overshoots in the motor speed when the classic tuning is used. Consequently, the online tuning response is much better than the classic tuning for both drive cycles. These overshoots are due to the overshoots of the motor torque as indicated in Figs. 9 and 10.

**Table 3:** EV parameters.

Parameter	Value
Kerb and passenger weight (m_EV_), kg	158
Frontal area (A_fa_), m^2^	0.875
Wheel radius (R_w_), m	0.23
Aerodynamic drag coefficient (C_ad_)	0.22
Gear ratio (G_ratio_)	2.5:1
*Tyre rolling resistance coefficient (C*_*tr*_ *)*	0.03

**Table 4:** Motor parameters.

Parameter	Value
poles (p)	4 poles
Nominal power	12 kW
Nominal Speed	1800 rpm
Base speed	5400 rpm
Nominal Torque	63.662 Nm
*R*_*s*_	0.389 Ω
*L*_*d1*_	2.7 mH
*L*_*q1*_	9.6 mH
*L*_*d3*_	1.1 mH
*L*_*q3*_	2 mH
*L*_*m13*_	0 mH
*λ*_*1 m*_	0.11 WbT
*λ*_*3 m*_	0.0012 WbT
Motor moment of inertia	0.0036 kg.m^2^
Total moment of inertia	0.14 kg.m2
Connection	Star

**Fig. 11:**
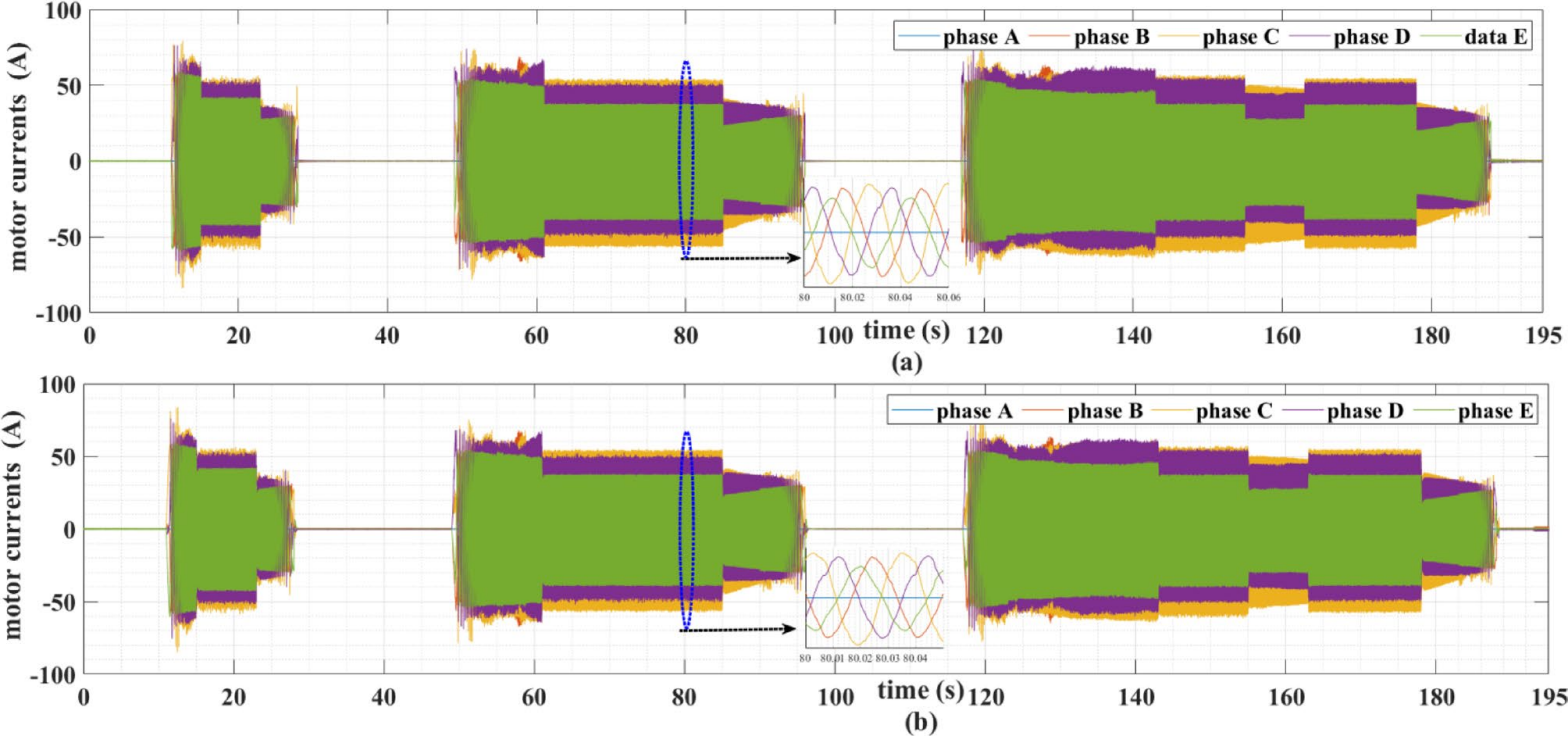
The PMSM currents for the ECE-15 drive cycles when (a) classic and (b) online PI tuning is used.

**Fig. 12:**
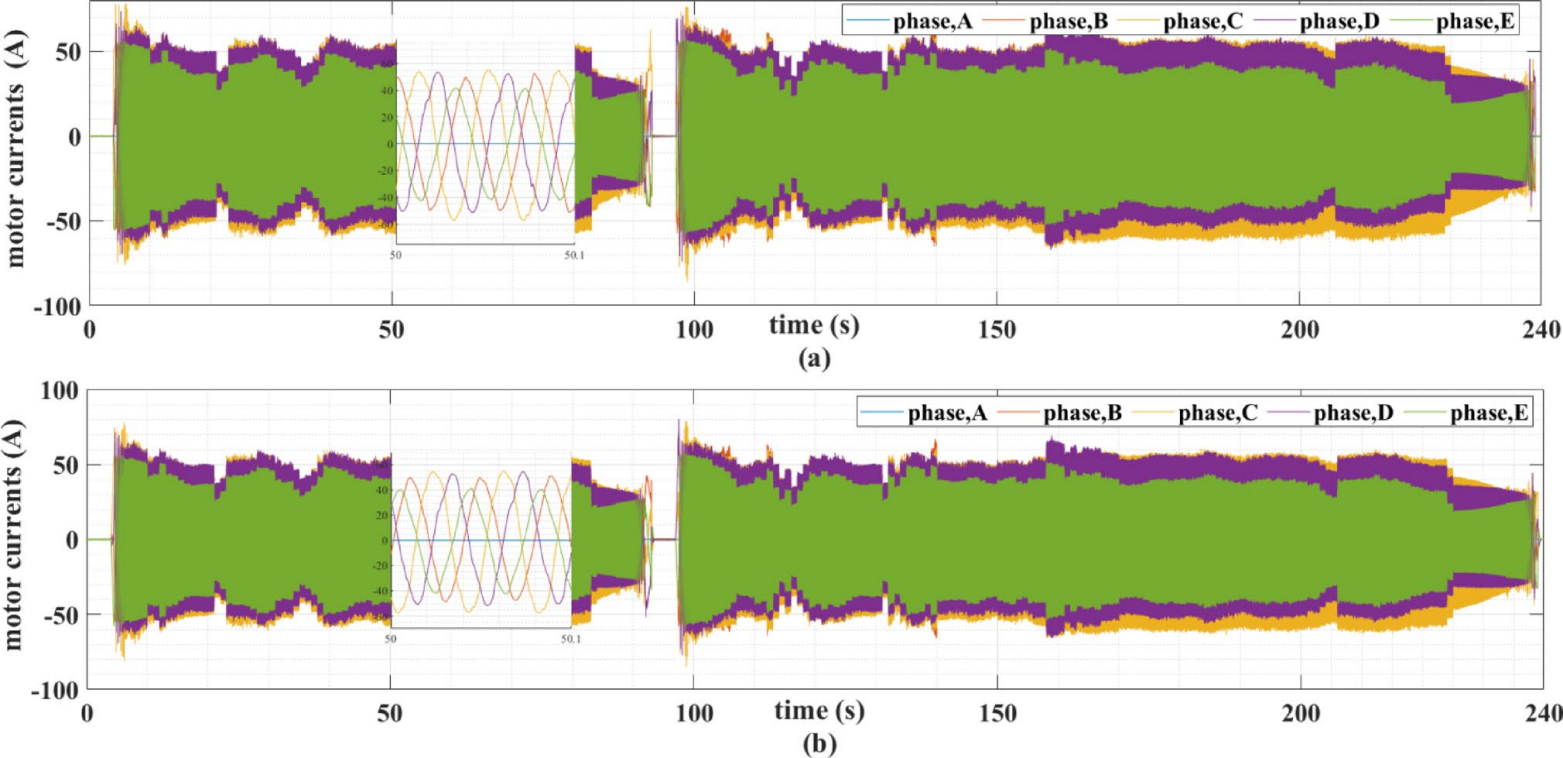
The PMSM currents for the custom IM240 drive cycle when (a) classic and (b) online PI tuning is used.

**Fig. 13:**
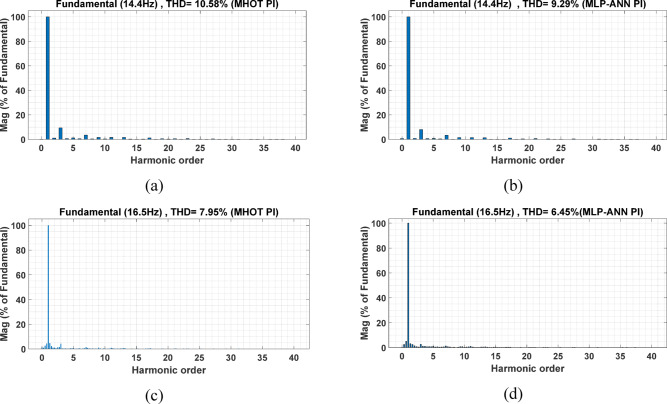
The PMSM currents harmonic spectrums: (a, b) ECE-15 and (c, d) Custom IM-240 drive cycles.

The PMSM currents are shown in Figs. 11 and 12. Note that the motor currents are almost sinusoidal. The harmonic spectra of these currents are shown in Fig. 13. It can be noticed from this figure that the currents’ THDs for MLP-ANN PI are less than those for MHOT PI for the two drive cycles.

**Fig. 14:**
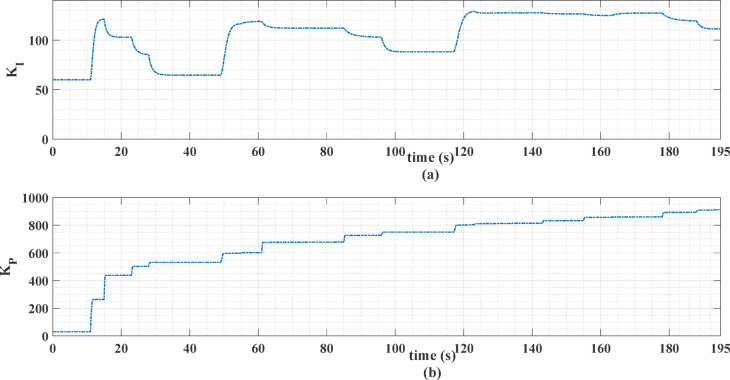
The Online PI gains for the ECE-15 drive cycle.

**Fig. 15:**
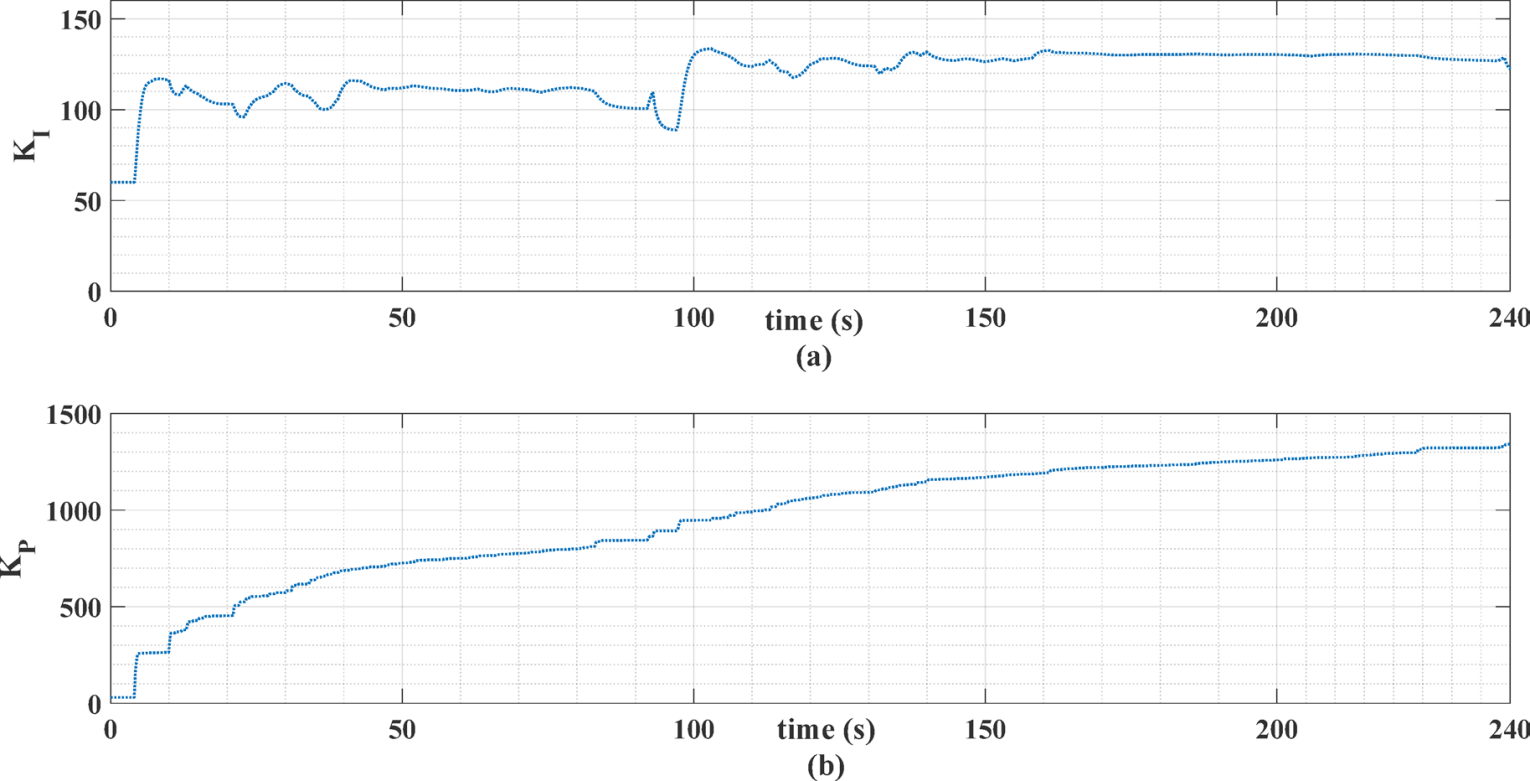
The online PI gains for the custom IM240 drive cycle.

The gains *K*_*p*_ and *K*_*i*_ of the PI controller are obtained with the MHOT, Honey Badger optimization technique [63] with a population size of 20, a maximum number of iterations of 50, and the standard control parameters as defined in^63^. These parameters are found to be 3.7858 and 7.868, respectively, when the ECE-15 drive cycle is used. The corresponding values are found to be 7.3438 and 5.0246 when the custom IM240 drive cycle is used. Figures 14 and 15 show the online MLP-ANN PI tuning gains for the two driving cycles.

**Fig. 16:**
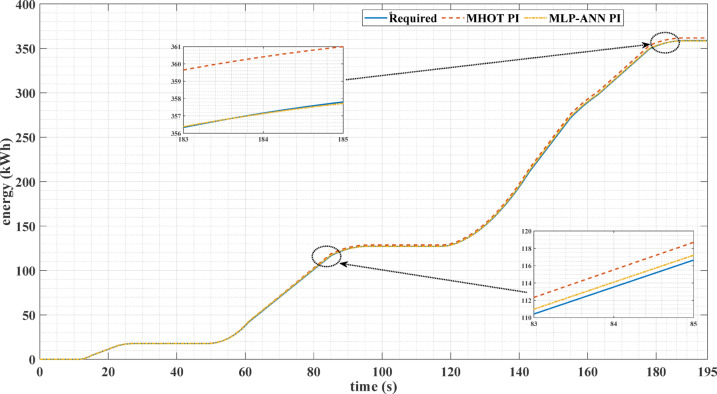
The required and actual EV energy consumption for the ECE-15 drive cycle when classic and online PI tuning are used.

**Fig. 17:**
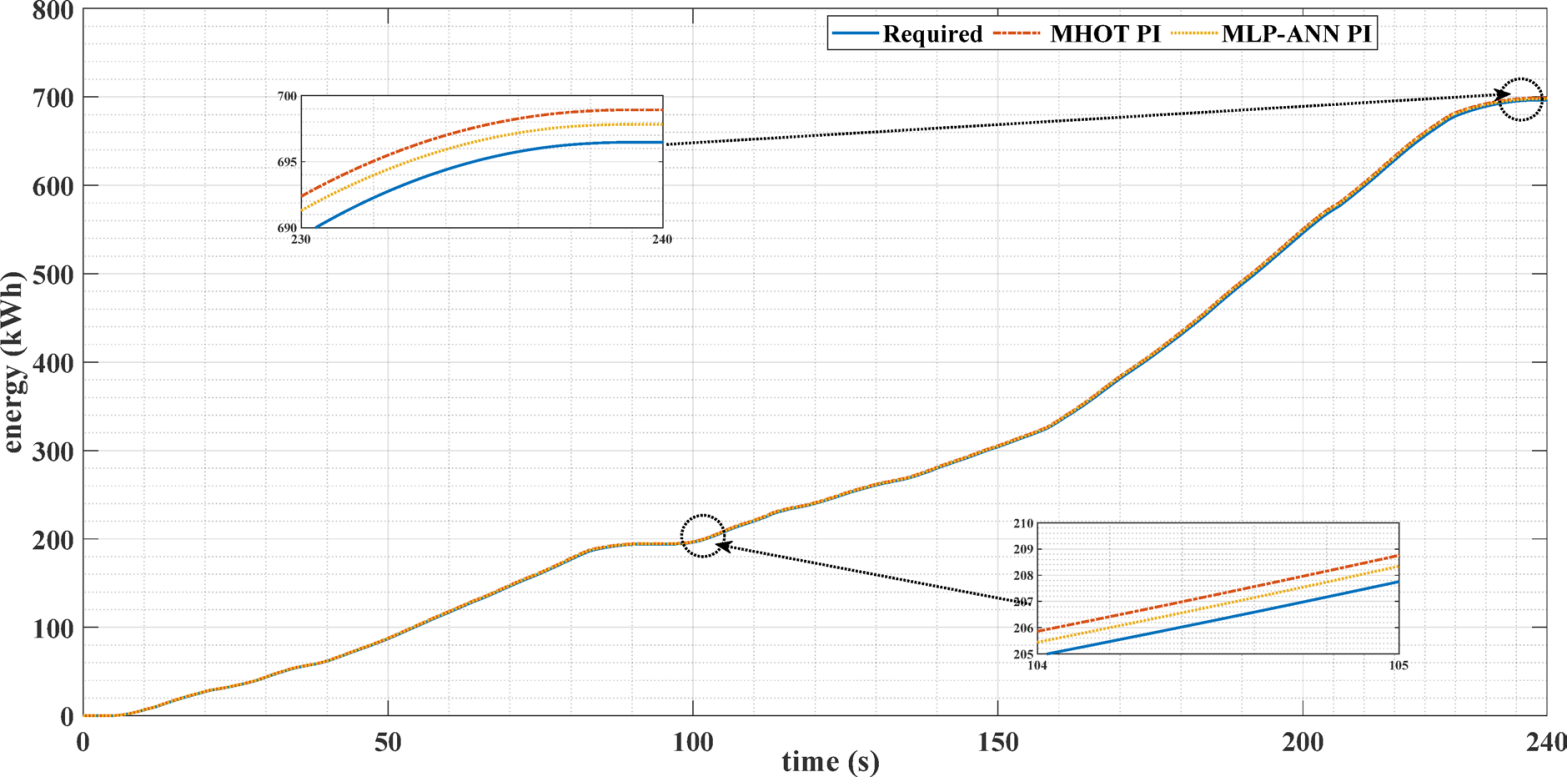
The required and actual EV energy consumption for the custom IM240 drive cycle, using both classic and online PI tuning.

Figures 16 and 17 show the required EV energy consumption and the actual energy when classic and online PI tuning are used for the two driving cycles. It can be noticed from these figures that the energy consumption for the online PI is less than that of the classic PI using the Honey Badger optimization technique.

Table 5 shows the comparison between the classic, Honey Badger, and online, MLP-ANN, tuning methods for the two drive cycles. It can be noticed from this table that online tuning achieves lower values of MSE, IAE, and percentage overshoot in the motor speed compared with the classic tuning method. Also, the torque ripples are less for the MLP-ANN tuning method. It can be noticed, also, from this table that the energy consumption for the MLP-ANN tuning is less than that of the classic PI using the Honey Badger optimization technique. Consequently, the use of the ANN online tuning for the PI controller attains energy savings.

**Table 5:** Comparison between the classic and MLP-ANN online tuning for the two different drive cycles.

Drive Cycle Type	ECE-15 drive cycle		Custom IM240 drive cycle	
Tuning approach of PI Controller	Honey Badger optimization technique (MHOT)	MLP-ANN Online tuning	Honey Badger optimization technique (MHOT)	MLP-ANN Online tuning
MSE	0.103627	0.001138	0.101279	0.000928
IAE	0.141322	0.002054	0.159901	0.001907
Percentage Overshoot (%)	6.985197	0.25989	3.573902	0.031759
Torque Ripples	9.5238%	7.30158%	7.0921%	6.55172%
EV required Energy, kWh	358.4726		696.4634	
Energy Consumption, Kwh	361.7488	358.6256	698.9165	697.8217
Energy Saving due to using MLP-ANN Online tunning, kWh	3.1232		1.0948	
%Energy Saving	0.87125%		0.1571%	

## Conclusion

This study presents a speed tracking control strategy for improving the performance of an electric vehicle drive system based 5-phase IPMSM under open phase fault. This method utilizes online tuning of a PI controller through a Multi-Layer Perceptron Artificial Neural Network (MLP-ANN). The integration of a 5-phase IPMSM and Model Predictive Control contributes to improved system reliability. The proposed approach is tested using the ECE-15 and custom IM240 drive cycles. Comparative analysis between the conventional PI tuning method based on Honey Badger Optimization technique and the MLP-ANN-based online tuning demonstrates superior performance of the latter. Specifically, the online tuning achieves lower Mean Square Error (MSE), Integral Absolute Error (IAE), and percentage overshoot in motor speed. For the ECE-15 drive cycle, the MSE, IAE, and overshoot percentage are 0.001138, 0.002054, and 0.25989%, respectively, while for the IM240 drive cycle, the values are 0.000928, 0.001907, and 0.031759%. The torque ripples, also, are reduced under fault using the presented MLP-ANN PI online tuning method compared to the classic MHOT PI control. Additionally, the online tuning method results in reduced energy consumption, with a maximum energy saving of 0.87125% compared to the conventional approach. These findings confirm that the proposed fault-tolerant control method enhances both speed response, torque ripples and energy efficiency under motor open-phase fault conditions. However, practical deployment must address challenges like fault detection accuracy, computational constraints, stability assurance, and safety certification.

As part of future work, the proposed control methodology will be implemented experimentally to validate its real-world performance.

## Data Availability

The data that support the findings of this study are available from the corresponding author upon reasonable request.
